# Potential Antiproliferative Activity and Evaluation of Essential Oil Composition of the Aerial Parts of* Tamarix aphylla* (L.) H.Karst.: A Wild Grown Medicinal Plant in Jordan

**DOI:** 10.1155/2018/9363868

**Published:** 2018-06-21

**Authors:** N. Alhourani, V. Kasabri, Y. Bustanji, R. Abbassi, M. Hudaib

**Affiliations:** ^1^School of Pharmacy, The University of Jordan, Amman, Jordan; ^2^Hamdi Mango Center for Scientific Research, The University of Jordan, Amman 11942, Jordan

## Abstract

Essential (volatile) oil from aerial parts of* Tamarix aphylla* (L.) H.Karst. (Tamaricaceae) grown wild in Jordan was hydrodistilled by Clevenger apparatus and analyzed by means of GC and GC-MS techniques.* In vitro* screening of potential cytotoxicity of the aqueous (AE) and ethanol (EE) extracts was also evaluated against human breast adenocarcinoma (MCF-7), colorectal adenocarcinoma (Caco-2), and pancreatic carcinoma (Panc-1) cancer cell lines as well as normal human fibroblasts. GC-MS analysis of* T. aphylla *EO revealed its richness in nonterpenoid nonaromatic hydrocarbons (52.39%), with predominance of 6,10,14-trimethyl-2-pentadecanone as the principal component. Biologically, the plant extracts exhibited cytotoxicity effects in dose-dependent manner against most of the tested cell lines, but potent effects were only predicted against MCF-7 cells with IC_50_ values of 2.17 ± 0.10 and 26.65 ± 3.09 *μ*g/mL for* T. aphylla* AE and EE, respectively.* T. aphylla* AE demonstrated a comparable cytotoxic effect with that offered by the control drug cisplatin (IC_50_ value of 1.17 ± 0.13 *μ*g/mL), even with higher safety profile against normal fibroblast cells (IC_50_ values of* T. aphylla* AE versus cisplatin: 79.99 ± 4.90 versus 9.08 ± 0.29 *μ*g/mL).* T. aphylla* extracts could be a valuable source for cytotoxic agents with high safety and selective cytotoxicity profiles. Unfortunately, no antiproliferative potential against Caco-2 or Panc-1 cancer cell lines was detected at a concentration less than 30 *μ*g/mL.

## 1. Introduction

Jordan's flora is rich in a wide variety of medicinal plants and hence is widely utilized by Jordanians for health maintenance. It is believed that the ease of access to herbal remedies alongside their wide safety margins encourages their usage by patients [1]. Recently, many efforts have been exerted to find out the potential roles of different medicinal plants in cancer treatment. Despite the fact that many of the widely grown plants in Jordan have been screened for their antiproliferative activities [2], many others either are under current investigation or are still unevaluated.

*Tamarix aphylla* (L.), which is known as ‘Tamarisk' or ‘Athel' in Jordan, is an evergreen tree with tiny, triangular, and scale-like leaves. It grows in dense groves and it flourishes in high alkalinity-salinity soils. Tamarisk is native throughout the Middle East across East, North, and Central Africa and to a little extent into parts of Western and Southern Asia [3]. Many communities have used* T. aphylla* in traditional medicine. Its leaves were used for wounds and abscess healing, as astringent, and for rheumatism and joint pain [4, 5]. Several studies have recognized the various types of secondary metabolites present in* T. aphylla*. Reports had identified the presence of flavonoids [6], phenolics [7], hydrolysable tannins [3], and alkaloids [7, 8] in the plant various extracts. Earlier investigations on the effects of* T. aphylla* on biological systems had revealed its insect growth inhibitory activity due to presence of ellagic acid [9]. The isolated isoferulic acid derivative, aphyllin, was also shown to exhibit a distinct radical scavenging activity and to improve the viability of human keratinocytes [10]. In Saudi Arabia, alcohol extract from leaves of* T. aphylla* was shown to possess antioxidant, anti-inflammatory, and wound-healing activities. The authors suggested that the presence of known active phytochemicals like flavonoids and polyphenols explains these reported effects [11]. The plant was also reported to exhibit analgesic and antipyretic activities [12].

Several previous studies have investigated the potential roles of various* T. aphylla* extracts in the prevention and/or treatment of many ailments [5, 11, 12]; nevertheless, only few researches have focused on investigating its volatile essential oil (EO) composition or evaluating the plant's antiproliferative effects against selected cancer cell lines. In this present study, the EO hydrodistilled from aerial parts of wild grown Jordanian species of* T. aphylla* was analyzed by gas chromatography-mass spectrometry (GC-MS) for purpose of chemical composition analysis.* In vitro* cytotoxic activities of the aqueous extract (AE) and ethanol extract (EE) against human colorectal adenocarcinoma (Caco-2), breast adenocarcinoma (MCF-7), and pancreatic carcinoma (Panc-1) cancer cell lines alongside normal human fibroblasts were also assessed. Interestingly, this is the first time that the EO composition and the cytotoxic activities of* T. aphylla* aerial parts are evaluated.

## 2. Materials and Methods

### 2.1. Phytochemical Analysis

#### 2.1.1. Plant Material

About 650 g of the aerial parts of* T. aphylla* (L.) H.Karst. was collected from north Amman in May 2016. Collected parts were taxonomically identified by Professor Khaled Tawaha (School of Pharmacy, the University of Jordan) and then set to air-dry under shade in a cool place for further study. Voucher specimen has been deposited in the Department of Pharmaceutical Sciences, School of Pharmacy, the University of Jordan, and given a specimen ID (TA-Hudaib-MAY16-001).

#### 2.1.2. Preparation of Crude Plant Extracts

Both the aqueous and ethanol extracts of the dried plant aerial parts were prepared by maceration. Separately, a powdered 100 g quantity was placed in round-bottomed flask and either a 1 L of 70% ethanol (EtOH) or 1 L distilled water was added in a ratio of 1:10. Flasks were kept for one week in a cool place; the filtrates were collected and then dried using rotary evaporator at 40°C. The dried extracts were kept in tightly closed flasks, at 4°C, for subsequent analysis.

#### 2.1.3. TLC

Thin layer chromatography (TLC) was performed (in duplicate) to obtain qualitative fingerprinting of the prepared crude extracts. TLC was achieved on precoated TLC silica gel plates (ALUGRAM SIL G/UV254, Macherey-Nagel GmbH & Co., Germany), using different mobile phases. Detection of chemical constituent was conducted as reported by Wagner and Bladt [14].

#### 2.1.4. Essential Oil Extraction

300 g of dried aerial parts of* T. aphylla* was soaked in 2.5 L of distilled water and then hydrodistilled for 2 hours using Clevenger-type apparatus. The hydrodistilled EO was collected using GC grade n-hexane, dried over anhydrous sodium sulphate, and then kept in tightly closed vial, at 4°C, until analysis.

#### 2.1.5. GC-MS Analysis

GC-MS analysis of EO was performed in duplicate after appropriate dilution of the hydrodistilled oil in GC grade n-hexane. Around 1 *μ*L aliquot of the diluted oil was injected into a split-splitless injector of a Varian Chrompack CP_3800 GC/MS/MS-200 (Saturn, Netherlands) GC-MS outfitted with DB-5 (5% diphenyl, 95% dimethyl polysiloxane) capillary column (30 m length x 0.25 mm ID, 0.25 *μ*m film thickness). The injector temperature was kept at 250°C with a split ratio of 1:10 and Helium was used as a carrier gas with a flow rate of 1 mL/minute. The column temperature was programmed to be initially isothermal at 60°C for 1 minute and then to increase up to 246°C at a rate of 3°C/minute and then to be kept isothermal at 246°C for 3 minutes for a total run time of about 66 min. The MS ionization source temperature was 180°C with an ionization voltage of 70 eV.

#### 2.1.6. GC-FID Analysis

Quantitative analysis of the hydrodistilled oil (performed in duplicate) was carried out using a Focus GC (Thermo Electron Corporation) gas chromatograph equipped with fused silica capillary column OPTIMA-5 (5% diphenyl 95% dimethyl polysiloxane; 30 m length × 0.25 mm ID, 0.25 *μ*m film thickness). The column was coupled to an injector (split-splitless type) and flame-ionization detector (FID). The same temperature program was used as mentioned above in GC-MS analysis section. Injector temperature was maintained at 250°C with split ratio of 1:50, while FID temperature was held at 300°C. Percent content (% w/w) of each component was calculated using its corresponding normalized relative area obtained by FID and assuming a unity response by all components [15].

#### 2.1.7. EO Component Identification

Qualitatively, identification of volatile components was carried out using built-in libraries (e.g., Wiley, Terpenes, NIST, and Adams' libraries). A comparison of the calculated Arithmetic Retention Index (RI) of each identified component with literature reference value measured with a column of identical polarity alongside MS spectrum matching helped to confirm the identification [15]. RIs of EO components were calculated relative to n-alkane hydrocarbons (C8-C20) analyzed under the same chromatographic conditions, as above, using the modified arithmetic equation by Van Den Dool and Kratz [16]. Quantitatively, the % content of each EO component was measured as mentioned above in GC-FID section.

### 2.2. *In Vitro* Cytotoxicity

#### 2.2.1. Cell Culture

Three different adherent cancer cell lines, named MCF-7 (ATCC: HTB-22™), Caco-2 (ATCC: HTB-37™), and Panc-1 (ATCC: CRL-1469™) cells, were used for testing the antiproliferative activity. Normal periodontal fibroblast cell line (provided from School of Dentistry, University of Jordan, Jordan) was used for testing selective toxicity of reference drugs and the different extracts. Cells were cultured in Dulbecco's Modified Eagle's Medium (DMEM, Caisson Laboratories Inc., USA) at 37°C. Cells dilution with medium to give optimal plating densities (determined by the supplier for each cell line) was carried out before they were plated in 96-well plates.

#### 2.2.2. Extracts and Reference Drugs Pretreatment for Cytotoxicity Assay

Each extract (10 mg) was weighed accurately and dissolved in 1 mL solvent. Solvents used were dimethyl sulfoxide (DMSO, tissue culture grade, Merck Schuchardt, Germany) for EE and DMEM for AE. Doxorubicin and cisplatin (both EBEWE Pharma GMBH Nfg. KG, Austria) were used as positive control drugs. Proper dilutions were made to achieve increasing concentrations of both extracts (0.1–800 *μ*g/mL) and positive controls (0.1–200 *μ*g/mL).

#### 2.2.3. Cytotoxicity Assay

Increasing concentrations of extracts and control drugs were added to the plated cells. Each concentration was added in 3 replicates for each test material and the test was repeated 3 times independently. The different concentrations used for EtOH extracts contain no more than 2% of solvent DMSO. As indicated in previous studies [17], plates were incubated for 72 hours. Once the exposure time had finished, cells growth was analyzed using tetrazolium reduction (MTT) assay as described by Riss [18]. Absorbance was read by multiwell plate reader (Bio-Tek Instrument, USA) at 570 nm using a reference wavelength of 630 nm.

#### 2.2.4. IC_50_ Value Calculation

As described by (1) and (2), percent cytotoxicity at each concentration was calculated from the obtained optical density (OD) by the plate reader. Before any further interpretation, all data were blank-adjusted. As described by (3), equations obtained from the logarithmic plot of % cytotoxicity versus concentration (*μ*g/mL) were used to calculate IC_50_.

(1) %  cells  viability=mean  OD  of  extract  wellsmean  OD  of  control  wells×100%

(2) %  Cytotoxicity=100%−%  cells  viability

(3) 50=slope×Ln  IC50+constant

## 3. Results

### 3.1. TLC

TLC tests revealed the presence of different secondary metabolites in tested* T. aphylla* extracts as shown in Table 1. TLC chromatograms pointed out the presence of flavonoids as the major components in both extracts. Minor fractions of terpenoids were only detected in the ethanol extract, while coumarins were presented in both extracts.

### 3.2. Oil Composition

**Figure 1:**
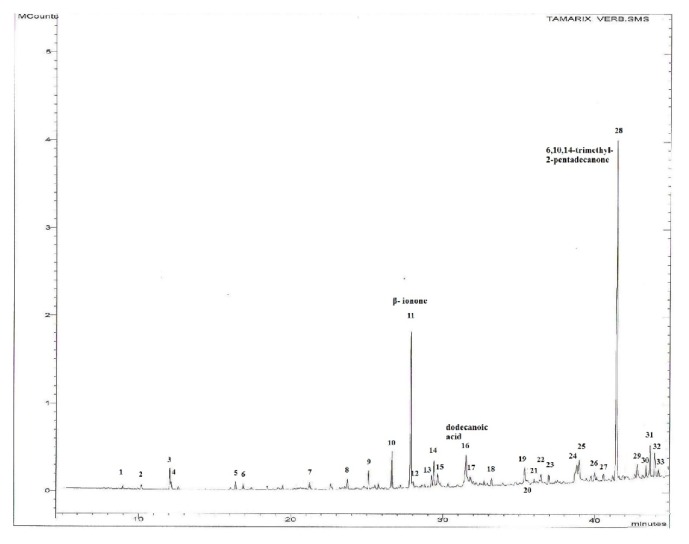
GC-MS chromatogram of the EO hydrodistilled from Jordanian* T. aphylla* growing in Amman (Jordan).

The GC-MS chromatogram of the hydrodistilled EO from* T. aphylla* aerial parts alongside the main identified components is shown in Figure 1. Many volatile principles that have been identified are presented in Table 2. As shown, the GC-MS analysis of* T. aphylla* EO resulted in the identification of 33 components predominated mainly by 6,10,14-trimethyl-2-pentadecanone as the principal component (32.39%) and *β*-ionone (13.74%) and dodecanoic acid (6.00%) as major ones. The majority of the volatile constituents which are classified as oxygenated nonterpenoid nonaromatic hydrocarbons (52.39%) were mainly containing, in addition to the principal component, dodecanoic acid (6.00%), tetradecanoic acid (3.35%), and tridecanal (2.39%). The prevalence of oxygenated sesquiterpenes is quite high (26.53%), with predominance of *β*-ionone (13.74%), 5E,9E-farnesyl acetone (2.82%), *α*-muurolen-15-al (2.27%), and 14-OH-9-epi-E-caryophyllene (2.16%). On the other hand, neryl acetone (2.82%) represented the main monoterpenes identified in* T. aphylla* essential oil.

### 3.3. Cytotoxicity Evaluation

**Figure 2:**
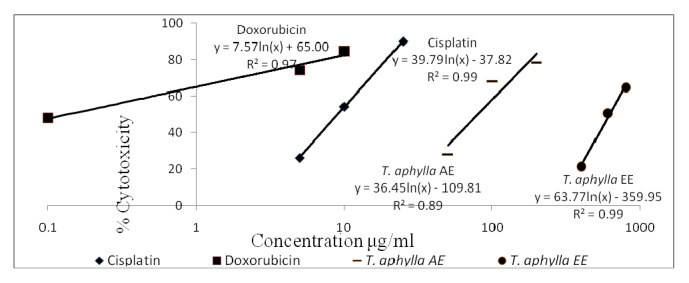
*In vitro* cytotoxic activity of cisplatin, doxorubicin, and* T. aphylla* aqueous extract (AE) and ethanol extract (EE) tested against normal fibroblast cells.

**Figure 3:**
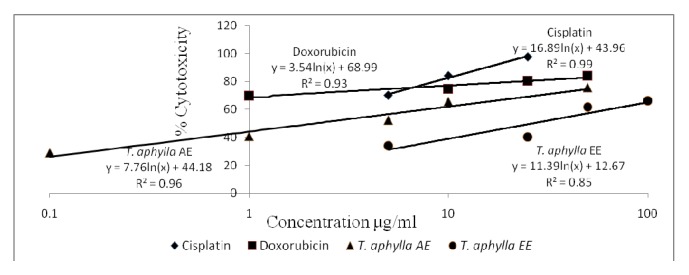
*In vitro* cytotoxic activity of cisplatin, doxorubicin, and* T. aphylla* aqueous extract (AE) and ethanol extract (EE) tested against Caco-2 colorectal cancer cell line.

**Figure 4:**
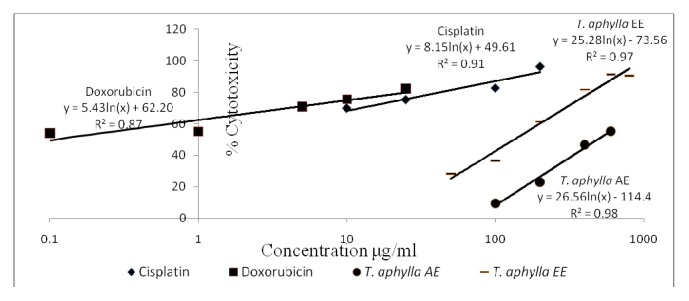
*In vitro* cytotoxic activity of cisplatin, doxorubicin, and* T. aphylla* EE tested against Panc-1 pancreatic cancer cell line. AE: aqueous extract; EE: ethanol extract.

**Figure 5:**
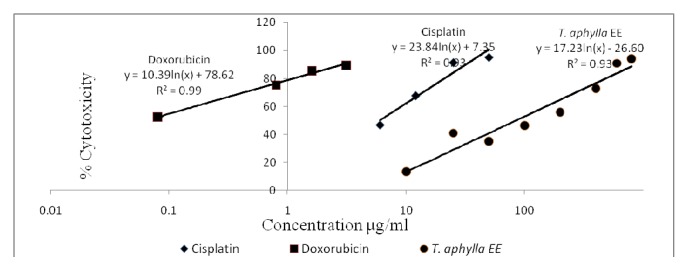
*In vitro* cytotoxic activity of cisplatin, doxorubicin, and* T. aphylla* aqueous extract (AE) and ethanol extract (EE) tested against MCF-7 breast cancer cell line.

Table 3 demonstrates the* in vitro* calculated IC_50_ values (*μ*g/mL) of* T. aphylla* AE and EE as well as control drugs (cisplatin and doxorubicin). The* in vitro* cytotoxicity profiles (% cytotoxicity versus concentration) of the control drugs alongside the different tested extracts are shown in Figures 2–5. Regarding the EO, the amount obtained by hydrodistillation unfortunately hindered its further biological evaluation due to presence in traces.

The American National Cancer Institute (NCI) guidelines set the limit of activity for crude plants extracts at 50% inhibition (IC_50_) of proliferation to be < 30 *μ*g/mL after the exposure time of 72 hours [19]. Accordingly, it appears that* T. aphylla* AE and EE are potentially potent cytotoxic extracts against MCF-7 cell line, with IC_50_ values (*μ*g/mL) of 2.17 ± 0.10 and 26.65 ± 3.09, respectively. Doxorubicin and cisplatin's respective IC_50_ values against MCF-7 cells are 0.01 ± 0.001 and 1.17 ± 0.13. Both extracts unfortunately lacked cytotoxic potential against Panc-1 or Caco-2 cell lines in the tested concentration range. Regarding the safety profile on normal fibroblasts, both extracts of* T. aphylla* demonstrated higher safety compared with doxorubicin and cisplatin. Figures 2–5 illustrate the different effects of the tested extracts and control drugs.

## 4. Discussion

Preliminary TLC test revealed the presence of flavonoids in both* T. aphylla* AE and EE, which was previously described by other researches [6]. The main identified volatile principle, 6,10,14-trimethyl-2-pentadecanone, is a nonaromatic oxygenated hydrocarbon (ketone) and has a slightly fatty aroma with reported antimicrobial [20] and antioxidant [21] properties. Other main identified compounds were *β*-ionone (13.74%) and dodecanoic acid (6.00%). Oxygenated sesquiterpenes account for almost 27% of the identified oil. Different compositions of the EO from different* Tamarix* species were also reported in literature. As described by Orfali [22], bicyclo[2.2.2]octan-2-one was found to be the major compound (46.09%) in* T. nilotica* of Saudi Arabia. Hexadecanoic acid methyl ester was reported as the major principle in* T. chinensis* fruit [23]. Hexadecanoic acid (in aerial parts and stems), 2,4-nonadienal (in flowers), and germacrene D (in leaves) were, however, reported as majors of* T. boveana *[24]. Like in* T. chinensis*, nonaromatic hydrocarbons resembled the abundant group of* T. aphylla* aerial parts, while fatty acids and fatty esters are the majors in* T. boveana* leaves [24]. On the other hand, among terpenes, oxygenated and hydrocarbon sesquiterpenes are prevalent in* T. aphylla* and* T. boveana* [24], respectively.

Biologically, several previous studies were also performed on the different species of* Tamarix*. In the current study, both the AE and EE of* T. aphylla* showed an interesting cytotoxic effect against MCF-7 cells. According to NCI guidelines [19], the selective cytotoxicity of* T. aphylla* AE (IC_50_ value of 2.17 ± 0.10 *μ*g/mL) against MCF-7 cells proved to be comparable to that of cisplatin (IC_50_ value of 1.17 ± 0.13 *μ*g/mL) and even with higher safety as revealed by their relative IC_50_ values on fibroblast cells (IC_50_ (*μ*g/mL): 79.99 ± 4.90 for AE versus 9.08 ± 0.29 for cisplatin). Despite the higher IC_50_ values on fibroblast cells compared with doxorubicin and cisplatin, both extracts unfortunately lacked cytotoxic potential against Panc-1 or Caco-2 cell lines in the tested concentration range.

On the other hand, recent study on methanol extract of* T. aphylla* had investigated its potential cytotoxicity using brine shrimp method and revealed 70% mortality rate at concentration of 500 *μ*g/ml [25]. In other studies, different extract of* T. nilotica* was tested against mouse lymphoma, rat hepatoma, and rat glioma cell lines. Interestingly, it was the presence of potent anticancer agents in the alcohol extract that could result in a powerful cytotoxic effect with % survival of 58.9 at 10 *μ*g/mL [22]. Also,* T. africana* shoot extract inhibited the growth of A-549 lung carcinoma cells, with an IC_50_ value of 34 *μ*g/mL [26]. Previous reports of phytochemical screening of* T. aphylla* revealed its high content of flavonoids, polyphenols, and tannins [3, 6, 8]. Several phenolic compounds isolated from Tamaricaceae were tested for their cytotoxic effects against different cancer cell lines and were shown to exhibit cytotoxic potentials [27]. Of these compounds are ferulic acid derivatives. Aphyllin, the isolated glycosylated isoferulic acid, exhibited a distinct radical scavenging activity and was found to improve the viability of human keratinocytes [10]. Ellagitannins were also reported to exhibit significant host-mediated antitumor and remarkable antiangiogenic activities [28]. Tamarixetin was also found to be cytotoxic against leukemia cells [29]. This latter compound inhibited proliferation in a concentration- and time-dependent manner, induced apoptosis, and blocked cell cycle progression. Syringic acid, a natural phenolic compound with selective antimitogenic activity on human malignant melanoma cells, was recently isolated from the methanol extract of* T. aucheriana *[30]. Methyl ferulate from* T. aucheriana* demonstrated cytotoxic and chemo-sensitizing effects [31], while glucuronosylated flavonoids from* T. gallica *aerial part were shown to prevent amyloid aggregation [32]. The potent cytotoxic activity observed for the tested extracts of* T. aphylla* under study is potentially attributed to presence of such compounds belonging principally to the phenolic fraction and other secondary metabolites as revealed by the TLC tests (Table 1).

## 5. Conclusion

The presence of different secondary metabolites with different biological activities renders medicinal plants valuable in drug discovery. Many biological activities have been linked to specific chemical entities present in the plants, such as flavonoids, tannins, polyphenols, and other compounds. The data obtained in this study suggest that different extracts of* T. aphylla* have potential antiproliferative activities against MCF-7 cancer cell line, which could be a source of promising lead compounds for the development of new treatments for some cancer types.

## Figures and Tables

**Table 1:** Major identified 2^ry^ metabolites' groups constituting the tested crude extracts of Jordanian *T. aphylla* as obtained by TLC analysis.

Sample	Flavonoids	Coumarins	Alkaloids	Terpenoids
*T. aphylla *AE	++	++	-* *-* *-* *-* *-	-* *-* *-* *-* *-* *-
*T. aphylla* EE	+++	+	-* *-* *-* *-* *-	+

AE: aqueous extract; EE: ethanol extract.

**Table 2:** Chemical composition of the EO hydrodistilled from Jordanian *T. aphylla* (Amman, Jordan) as analyzed by GC-MS.

No.	Rt.^a^	RI Lit.^b^	RI Exp.^c^	Compound	% content^d^
1	8.971	1022	1021	*o-*cymene	Tr.^e^
2	10.204	1054	1053	*γ*-terpinene	0.37
3	12.069	1099	1102	cis-decahydronaphthalene	1.44
4	12.152	1101	1104	*α*-thujone	0.57
5	16.369	1200	1203	cis-4-caranone	0.63
6	16.899	1217	1215	*β*-cyclocitral	0.31
7	21.284	1315	1316	2E,4E-decadienal	0.67
8	23.757	1373	1374	*β*-E-damascenone	0.81
9	25.099	1408	1406	dodecanal	1.35
10	26.619	1444	1443	neryl acetone	2.82
**11**	**27.856**	**1477**	**1473**	**β** **-ionone**	**13.74**
12	28.051	1489	1478	cis-eudesma-6,11-diene	0.32
13	29.251	1508	1508	farenal	1.02
14	29.399	1509	1512	tridecanal	2.39
15	29.656	1521	1518	*β*-sesquiphellandrene	1.20
**16**	**31.423**	**1565**	**1563**	**dodecanoic acid**	**6.00**
17	31.807	1579	1573	n-hexyl benzoate	1.69
18	33.196	1611	1609	tetradecanal	0.75
19	35.324	1668	1666	14-OH-9-epi-E-caryophyllene	2.16
20	35.567	1672	1673	5-isocedranol	0.81
21	36.01	1685	1685	ishwarone	0.76
22	36.454	1700	1697	n-heptadecane	0.92
23	36.972	1713	1711	2E,6Z-farnesal	0.74
24	38.818	NA^f^	1763	tetradecanoic acid	3.35
25	38.971	1767	1768	*α*-muurolen-15-al	2.27
26	39.996	1800	1797	octadecane	0.97
27	40.561	1816	1813	2E,6E-farnesoic acid	0.57
**28**	**41.424**	**1845** ^**g**^	**1839**	**6,10,14-trimethyl-2-pentadecanone**	**32.39**
29	42.776	1886	1879	5E,9Z-farnesyl acetone	1.64
30	43.381	1900	1897	nonadecane	1.07
31	43.624	1913	1904	5E,9E-farnesyl acetone	2.82
32	43.932	NA	1913	14Z-Methyl-8-hexadecenal	2.48
33	44.177	1921	1921	methyl hexadecanoate	0.72
					
			Monoterpenes (MT)		7.62
			Hydrocarbon MT: No. 1-3		1.81
			Oxygenated MT: No. 4-8,10		5.81
			Sesquiterpenes (ST)		28.05
			Hydrocarbon ST: No. 12,15		1.52
			Oxygenated ST: No. 11,13,19-21,23,25,27,29,31		26.53
			Nonterpenoid nonaromatic compounds: No. 9,14,16,18,22,24,26,28,30,32,33		52.39
			Nonterpenoid aromatic compounds: No. 17		1.69
			Total Identified %		89.75

Notes: compounds are listed in order of their elution times from a DP-5 column. ^a^: retention time; ^b^: literature RI^13^; ^c^: experimental RI relative to (C8-C20) n-alkanes; ^d^: the percentage composition based on peaks areas; ^e^: traces: below 0.1 % content; ^f^: RI value not available in literature; ^h^: reference [13]. Compounds in bold are the major (≥ 4%).

**Table 3:** Cytotoxicity IC_50_ values (mean ± standard deviation (SD)) of cisplatin, doxorubicin, and *T. aphylla* extracts tested in a panel of cancer cell lines.

Treatment		Cytotoxicity (IC_50_ value: mean ± SD; *μ*g/mL)			
		MCF-7	Caco-2	Panc-1	PDL Fibroblasts
Doxorubicin		0.01 ± 0.001	0.10 ± 0.01	0.06 ± 0.01	0.14 ± 0.02
Cisplatin		1.17 ± 0.13	1.11 ± 0.15	5.97 ± 0.57	9.08 ± 0.29
*T. aphylla* AE		2.17 ± 0.10	479.76 ± 54.99	Nontoxic^*∗*^	79.99 ± 4.90
*T. aphylla* EE		26.65 ± 3.09	130.55 ± 12.25	88.74 ± 2.44	154.90 ± 3.29

*Notes.* ^*∗*^ *Nontoxic within the investigated concentration range (0.1–800 μg/mL). AE: aqueous extract; EE: ethanol extract.*

## Data Availability

The data used to support the findings of this study are available from the corresponding author upon request.
